# Influencing the exposome, patient macroenvironment, tumor microenvironment through-line to improve clinical outcomes

**DOI:** 10.3389/fonc.2026.1929067

**Published:** 2026-10-12

**Authors:** Samuel F. Yanuck, Neil E. Alexis

**Affiliations:** 1 Program on Integrative Medicine, Department of Physical Medicine and Rehabilitation, University of North Carolina School of Medicine, Chapel Hill, NC, United States; 2 Center for Environmental Medicine, Asthma, and Lung Biology, Division of Allergy and Immunology, University of North Carolina at Chapel Hill, Chapel Hill, NC, United States

**Keywords:** exposome, oncometabolites, patient macroenvironment, T cell polarization, T cell infiltration, tumor immune microenvironment, tumor immunity, tumor microenvironment

## Abstract

Previous studies have identified an association between a tumor microenvironment characterized by a predominance of T helper cell type 2 (Th2) system cells (Th2 cells, M2 macrophages, innate lymphoid cells type 2), regulatory T cells, and myeloid derived suppressor cells with poorer cancer prognosis. In contrast, a tumor microenvironment characterized by Th1 system cells (Th1 cells, M1 macrophages, natural killer cells, CD8+ cells, innate lymphoid cells type 1) is associated with a more favorable prognosis. T2-high asthma, a quintessential Th2-dominance-based disease, is associated epidemiologically with more than double the incidence of lung, breast, prostate, and colon cancers, suggesting a connection between the Th2 immune dominant patient macroenvironment and the Th2 immune dominant tumor microenvironment. Stress, air pollution, toxicants, sleep disruption, obesity, and many other influences from the exposome are known to drive the patient into Th2 dominance. Thus, the authors hypothesize that there is a through-line connecting the exposome, patient macroenvironment, and tumor microenvironment. With this context in mind, specific defects in the tricarboxylic acid (TCA) cycle produce oncometabolites that drive oncogenic development in tissues. However these same TCA cycle defects drive macrophages to the M1 phenotype, which activates the Th1 system. Th1 system cells are responsible for clearance of oncogenically transformed cells. This should represent a check against the oncogenic influence of the oncometabolites created by the TCA cycle breaks. The authors hypothesize that, in Th2 dominant patients, the TCA cycle breaks fail to shift macrophages to the M1 phenotype, the Th1 system fails to upregulate, and the normal check against oncogenic development fails to occur. If confirmed by subsequent studies, this model may help explain the more than doubled incidence of common cancers in asthma and potentially other Th2 atopic diseases. The authors discuss clinical implications and approaches for operationalizing existing knowledge of the exposome and its impact on patient immunology.

## Graphical abstract

**Figure:**
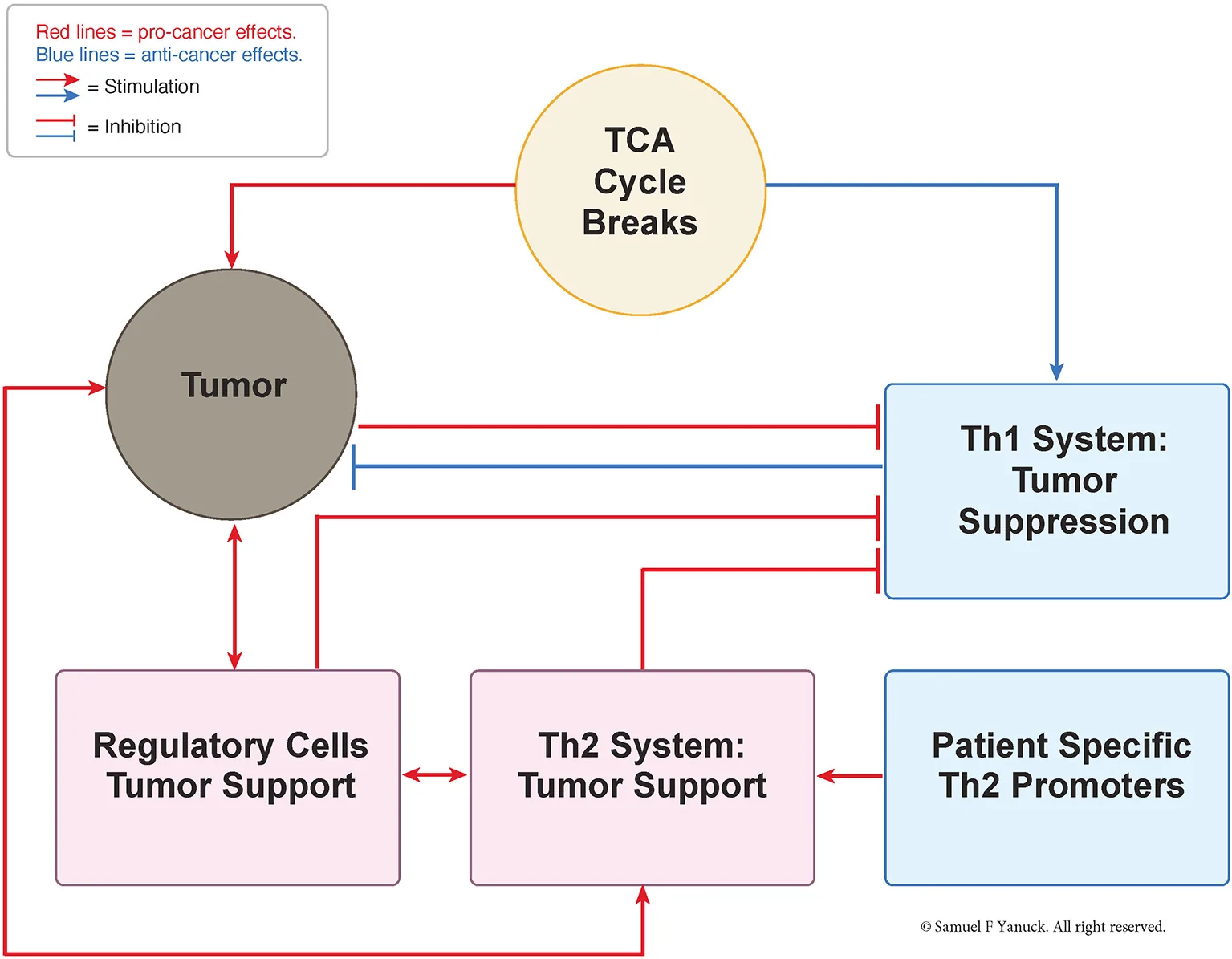

## 1 Introduction

When the existing research on the tumor microenvironment (TME), the patient macroenvironment (PMaE), exposomic influences, and the effects of TCA cycle breaks are combined, a picture emerges that forms the background for the authors’ hypothesis. The emerging picture contributes to the understanding of the substantially increased risk of colon, breast, prostate, and lung cancer in asthma patients and also presents potential opportunities for clinical intervention, with the goal of improving clinical outcomes.

The TME is composed of tumor cells, stromal cells, immune cells, cytokines, and other signaling molecules (1). The TME whose composition includes a predominance of T helper cells type 1 (Th1) system cells, crucially including M1 macrophages, is associated with more favorable prognosis. The TME whose composition includes a predominance of Th2 system cells, crucially including M2 macrophages, as well as regulatory T cells (Tregs) and myeloid derived suppressor cells (MDSCs) is associated with a more unfavorable prognosis (2–12). Thus, there is a connection between the immunological status of the TME and patient outcomes.

T2-high asthma is a prototypical Th2-dominance-based disease. Patients with asthma have substantially more than double the rates of colon, lung, breast, and prostate cancer (13, 14). This and other research discussed below suggest a strong connection between the whole-body immunological environment, which we term the patient macroenvironment (PMaE), and the patient’s TME (15, 16). This research supports an association between the PMaE, the TME, and thus patient outcomes.

A wide range of exposomic influences have been shown to shift the PMaE toward Th2. The authors hypothesize that this Th2 promoting influence of the exposome on the PMaE carries through to the TME, such that there is a through-line connecting the exposome, patient macroenvironment, and tumor microenvironment. Many common elements of the exposome have Th2-promoting immunological effects, including dysregulation of the microbiome (17), stress chemistry (18–23), sleep dysregulation (24, 25), air pollution (26), and other exposomic elements discussed below.

This immunological context forms a background, into which the impacts of tricarboxylic acid (TCA) cycle dysregulation can be considered. The TCA cycle (aka Kreb’s cycle) is a central hub of cell metabolism. Breaks and/or mutations in specific TCA cycle enzymes generate metabolites that have known metabolic and immunological effects that oppose each other in their influences on oncogenic transformation in tissues.

Breaks and/or mutations in TCA cycle enzymes (TCA breaks) generate oncogenic metabolites that drive the formation of cancer cells in the tissues in which the TCA breaks occur (27, 28).

When TCA breaks at isocitrate dehydrogenase (IDH), fumarate hydratase (FH), and succinate dehydrogenase (SDH) occur in macrophages, including in tumor associated macrophages (TAMs), the TAMs polarize to the M1 phenotype (11, 12, 29). M1 macrophages make IL-12, polarizing naïve T cells to the Th1 phenotype, solidifying lineage commitment of existing Th1 cells, and increasing Th1 cell production of interferon gamma (IFNγ) (30–33). This Th1 system upregulation increases the anti-tumor effects that eliminate oncogenically transformed cells, reducing cancer risk in patients who do not yet have cancer, and improving prognosis in cancer patients, by shifting the TME away from Th2 dominance, toward the Th1 dominant TME associated with better prognosis (2–10).

TCA breaks can occur in macrophages undergoing reprogramming in response to toll-like receptor 4 (TLR4) stimulation by signals like lipopolysaccharide (LPS) or inflammatory cytokines in the tissue environment (34). Separately, macrophage metabolism can also be influenced toward a higher incidence of TCA breaks by dysregulation of whole-body metabolic processes (35–37). In this way, an increase in TCA breaks occurring in tissue as a result of whole-body influences such as metabolic dysregulation can affect parenchymal tissue cells as well as tissue-resident and monocyte-derived macrophages.

The authors hypothesize that, when TCA breaks occur in the normal patient, the oncogenic effects and M1 polarizing effects of the TCA breaks oppose each other, with the M1 macrophages driving Th1 system activation, favoring elimination of oncogenically transformed cells. However, in the patient whose PMaE is Th2 dominant, Th1 system activation is blunted, Th1 system-based clearance of oncogenically transformed cells is diminished, and immunological conditions consistent with tumor development and progression are favored. This is consistent with evidence suggesting that patients with Th2 dominant comorbidities like asthma have more than double the risk of colon, lung, breast, and prostate cancer.

The authors further hypothesize that future studies using clinical applications aimed at influencing the immunological status of the PMaE (by changing exposomic elements, for example) will yield a favorable influence on the TME and thus favorably influence patient prognosis.

The following sections describe the TME, PMaE, Exposomic effects, and TCA break effects, followed by a discussion of the picture formed from the through-line integration of these elements and the clinical implications of the integrated picture.

## 2 The tumor microenvironment

The tumor microenvironment (TME) is composed of the tumor cells, immune cells, cytokines, and other elements in the tumor and surrounding tissues (1). Emerging evidence suggests additional contributions to the TME from hypoxia, metabolic characteristics, extracellular matrix, and pH (38). In the TME, different CD4+ T cell phenotypes, and the associated immune cells and cytokines for each phenotype, have significant influences on tumor proliferation, survival and ultimately, cancer outcomes (2–10). The studies described in this section establish that influencing the TME away from Th2 and Treg dominance and toward Th1 dominance yields better prognosis.

### 2.1 Immune cells in the tumor microenvironment

#### 2.1.1 The Th1 system

The Th1 system of cells includes Th1 cells, M1 macrophages, Natural Killer (NK) cells, innate lymphoid cells type 1 (ILC1’s), and CD8+ cytotoxic T lymphocytes (CD8 cells, or CTL’s). M1 macrophages generate interleukin-12 (IL-12), which activates Th1 cells, NK cells, and CD8+ T cells. These in turn generate IFNγ, which activates M1 macrophages. In this fashion, the cells of the Th1 system activate each other and increase Th1 system activation overall. The effect of this activation is to forward the functions associated with the Th1 system, including anti-tumor effects (10, 39). The IFNγ made by Th1 cells, NK cells, and CD8+ T cells also inhibits differentiation, proliferation, and cytokine production by Th2 cells (40, 41).

In human trials, TME’s with greater numbers of Th1 cells were associated with positive responses to several types of treatments. These included responders to anti-programmed death-1 (PD-1) antibody therapy (42), ipilimumab and interferon alpha therapy in malignant melanoma (43), pembrolizumab in gastric cancers (44), chemoradiation followed by surgery in locally advanced esophageal squamous cell carcinoma (45), and chemotherapy in high grade serous ovarian cancer (46). Greater numbers of Th1 cells were also associated with improved survival in prostate cancer, reviewed in (47).

#### 2.1.2 The Th2 system

The Th2 system of cells includes Th2 cells, M2 macrophages, eosinophils, mast cells, basophils and innate lymphoid cells type 2 (ILC2’s). Th2 system cells interact with tumor cells in a mutually reinforcing pattern (10, 48). Th2 cells and ILC2’s make interleukin-4 (IL-4) that inhibits Th1 cell differentiation, downregulates Th1 cell production of IFNγ, and inhibits Th1 cell trafficking into tissue. Thus, the Th1 and Th2 systems are known to inhibit each other’s activation and functions (40, 41, 49, 50).

In human trials, TME’s with greater numbers of Th2 cells were associated with poorer prognosis in melanoma (43), in pancreatic cancer (51), in high grade serous ovarian cancer (52) and were associated with aggressive features in estrogen receptor-positive breast cancer (53). Nevala, et al. identified patients with metastatic melanoma as ‘exist[ing] in a state of Th2-mediated “chronic inflammation…”‘ (54). Tumor infiltration with Th2 cells was associated with tumor recurrence in head and neck squamous cell carcinoma (55).

#### 2.1.3 Non-conforming treatment-related settings

Though the literature describes a consistently strong association between the Th1-dominant and Th2-dominant TME with favorable and unfavorable outcomes respectively, including in human trials discussed here (42–47, 51–55), there are specific clinical settings in which the immunological details of the disease-treatment interface function differently. In acute lymphoblastic leukemia (ALL), 50% of patients relapse within the first year after CAR T cell therapy (56). Bai, et al. reported that the addition of IL-4 to CAR T cells during antigen-specific activation “alleviates CAR T cell dysfunction” and that “elevated type 2 functionality in CAR T infusion products is significantly associated with patients maintaining a median B cell aplasia duration of 8.4 years” (57). A Th2 dominant TME is associated with higher proliferation rates (5–7, 10). Tumors with higher proliferation rates are more vulnerable to anti-mitotic and cytotoxic medications. This has been described, for example, in estrogen receptor positive breast cancer (53) and in high-grade neuroendocrine neoplasms (58). IL-5 (produced by Th2 cells) stimulates eosinophil expansion and tumor infiltration, improving the response to immune checkpoint blockade in breast cancer patients (59). Thus, it is important to evaluate the extent to which the model described by the authors applies or does not apply to a particular cancer type, stage, or grade and to a particular treatment type.

Th2 cells have also been shown in a murine model to inhibit colon and pancreas tumors (60). Evidence that Th2 cells are associated with a mix of effects, some of which may contribute to antitumor immunity in some circumstances, has been reviewed by Schreiber, et al. (61).

#### 2.1.4 Immunosuppressive cells

In the TME, cells that suppress the anti-tumor immune response include Tregs, MDSC’s, tumor-associated macrophages (TAM’s) that have polarized to the M2 phenotype, and senescent (exhausted) effector T cells. Along with Th2 system cells, these cells exchange cytokines and other signaling molecules with tumor cells to support and reinforce tumor instantiation and progression (6, 10, 62).

The TME characterized by a predominance of Tregs, MDSC’s, Th2 system cells, and neutrophils, is typically associated with poor prognosis, including tumor growth and greater likelihood of metastasis (6, 10, 62). The cytokine-mediated crosstalk between the tumor and cells of the Th2 and Treg systems, as well as crosstalk between Th2 system cells and regulatory cells, has been described (6, 10, 62) and is represented in **Figure 1**.

**Figure 1:**
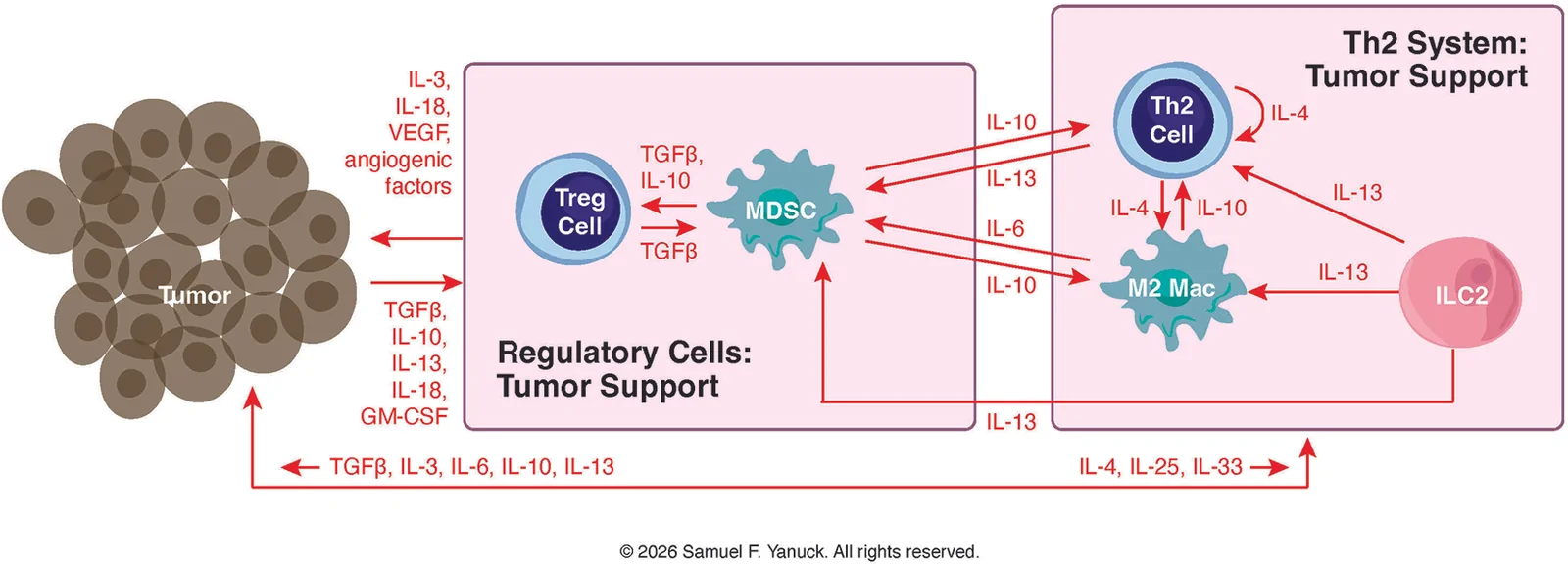
Interactions between Th2 system cells, immune-suppressive Tregs and MDSCs, and tumor cells. Cells of the Th2 and regulatory systems signal each other and engage in bi-directional reinforcing signaling with the tumor, supporting tumor progression. GM-CSF, granulocyte-macrophage colony-stimulating factor; IL, interleukin; ILC, innate lymphoid cell; mac, macrophage; MDSC, myeloid-derived suppressor cell; TGF, transforming growth factor; Th, T helper cell; Treg, regulatory T cell; VEGF, vascular endothelial growth factor.

In contrast, the TME characterized by a predominance of cells of the Th1 system is typically associated with better prognosis, including inhibition of tumor growth and decreased likelihood of metastasis (2–10). Previous TME studies include reviews of the role the TME plays in cancer prevalence and incidence overall (2–10), as well as studies of specific cell types within the TME in multiple types of cancer. These include improved prognosis with greater numbers of M1 macrophages (63), CD8+ T cells (6), NK cells (64, 65), and ILC1’s (66–68). Immune cell infiltrates in the TME have been studied with the same conclusions by specific cancer type, for example, in bladder (69), lung (70, 71), prostate (72), and melanoma (54, 73–75).

Signaling mechanisms by which tumor cells (TCs) and immune cells influence each other in both Th1 dominant (tumor-surveillant) and Th2 dominant (tumor permissive/promoting) TME’s have been described (2–4, 76, 77). **Figure 2** shows key features of the TME characterized by successful anti-tumor immunity vs. tumor – immune cell crosstalk associated with tumor progression. The right side of **Figure 2** shows reinforcing crosstalk between tumor cells and cells of the Th2 and regulatory systems. The left side of the diagram shows the anti-cancer surveillance mechanisms driven by cells of the Th1 system. The center of the diagram shows key reciprocal inhibition mechanisms between cells of the Th1 system, cells of the Th2 and regulatory (tolerogenic) systems, and tumor cells (**Figure 2**).

**Figure 2:**
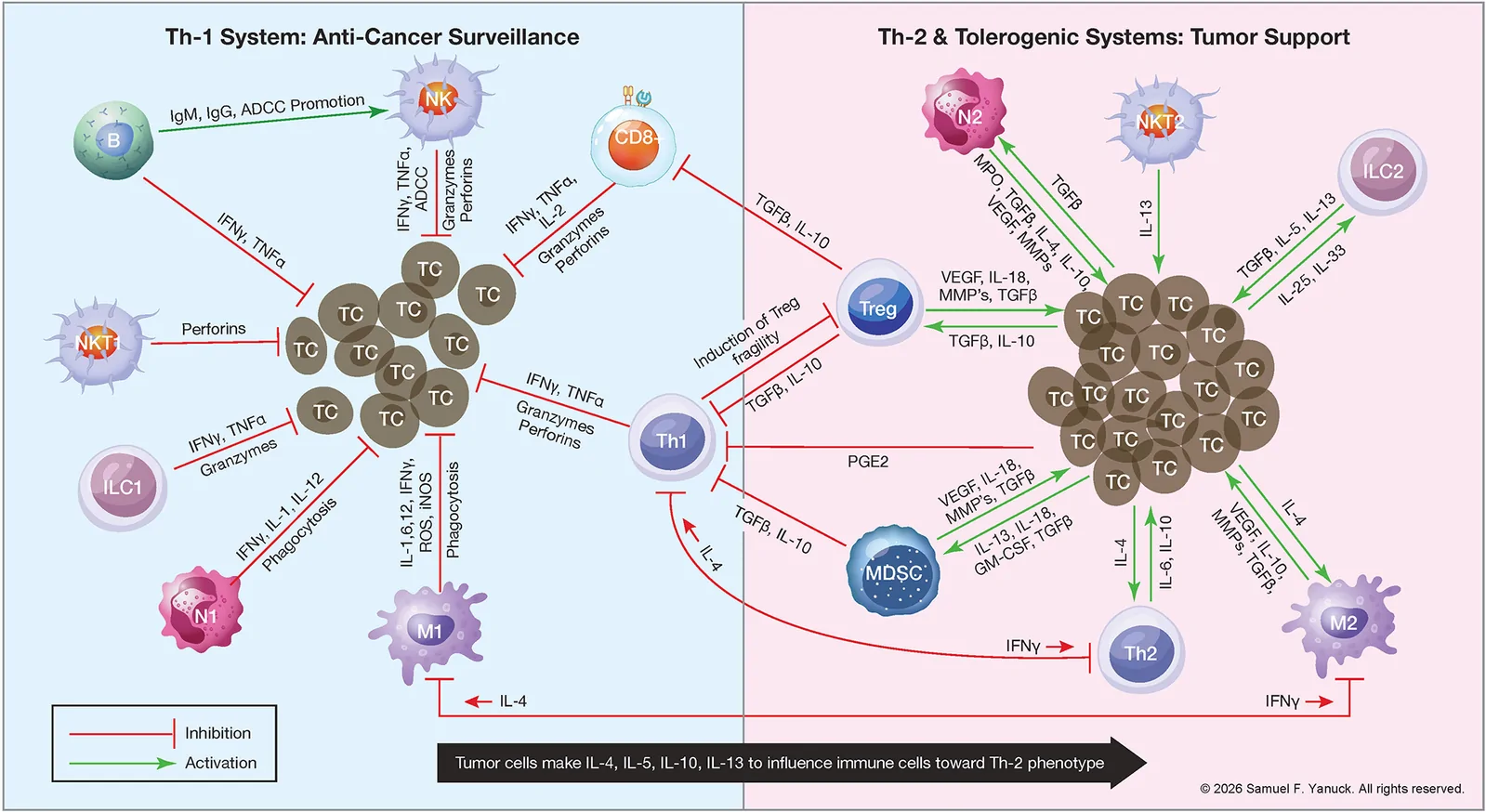
The tumor microenvironment – (I) The left side shows the TME with successful anti-cancer immune surveillance. Th1 system cells signal each other using Th1-associated cytokines and carry out a tumor-suppressive immunological program. (II) The right side shows the TME with tumor-immune cell crosstalk that favors the tumor. (III) Cells of the Th1 and Th2/Regulatory systems inhibit each other’s programs, creating a complex equation that determines the patient’s TME immunological status. ADCC, antibody-dependent cellular cytotoxicity; B, B cell; GM-CSF, granulocyte-macrophage colony-stimulating factor; IFN, interferon; Ig, immunoglobulin; IL, interleukin; ILC, innate lymphoid cell; iNOS, inducible nitric oxide synthase; M, macrophage; MDSC, myeloid-derived suppressor cell; MMP, matrix metalloproteinase; MPO, myeloperoxidase; N, neutrophil; NK, natural killer cell; NKT, natural killer T cell; PGE2, prostaglandin E2; ROS, reactive oxygen species; TC, tumor cell; TGF, transforming growth factor; Th, T helper cell; TNF, tumor necrosis factor; Treg, regulatory T cell; VEGF, vascular endothelial growth factor.

Increased numbers of CD8+ T cells (a Th1 system cell) in the TME have been associated with favorable prognosis across many cancer types (4–6, 78). There are exceptions, including clear cell renal cell carcinoma, prostate cancer, follicular lymphoma, and Hodgkin lymphoma, in which greater numbers of CD8+ T cells in the invasive margins of tumors is associated with shorter progression-free survival (PFS), due to the presence of polyclonal CD8+ T cells with reduced cytotoxic capacity (6). A study by Gentles et al. (79) showed that while tumor infiltration by CD8+ T cells was associated with good prognosis, tumor infiltration with tumor associated macrophages (TAMs), eosinophils, and neutrophils was associated with poor prognosis.

The strong connection between CD8+ cell numbers in the TME and positive prognosis has led to development of the Immunoscore test, a quantitative assessment of CD3+ and CD8+ cell numbers in tumor slices. The Immunoscore is a quantitative tumor assay that uses an immunohistochemistry-based digital process to analyze tumor sections from the tumor core and invasive margins. This replaces visual inspection by a pathologist. An analytical score is reported for the extent of CD3+ and CD8+ T cells in the TME of tumor samples, with greater numbers of both CD3+ and CD8+ T cells in the tumor correlating with better prognosis. The Immunoscore assay has been validated for colon cancer prognostic assessment, with greater CD8+ T cell infiltration established to be a favorable prognostic marker. The Immunoscore test has also been introduced into the World Health Organization classification of digestive tumors (80–82).

### 2.2 Reciprocal inhibition between T cell types in the TME

#### 2.2.1 Th1 and Th2 systems cells in the TME

The reciprocal inhibition between cells of the Th1 and Th2 systems is present in all tissue compartments and is crucial to understand in the TME, since the outcome of this reciprocal inhibition equation in the TME strongly influences the success or failure of tumor surveillance mechanisms in the TME and thus patient outcome.

Direct signaling interactions between immune cells and tumor cells play an important role in determining the immunological status of the TME. Direct interactions between cells of the Th1 and Th2 systems are also essential in determining whether a preponderance of the immune cells in the TME will be Th1 system cells or Th2 system cells. This has a potentially determinative influence on the outcome of immune cell – tumor cell interactions. The studies in this section establish that there is a reciprocal inhibition between Th1 immunity and Th2 immunity. This is relevant to the authors’ hypothesis because it suggests that activation of the patient’s Th1 immunity may be an important component of an effective strategy for influencing the TME away from Th2/Treg co-dominance.

Reciprocal inhibition between the Th1 and Th2 systems is long established (49, 50), with each system suppressing cell numbers and activity of the other system via competitive influence on naïve T cell polarization, and via cytokine mediated inhibition of the other’s effector cytokine expression (41, 66, 83–85). Though these immunological mechanisms are well established in the literature, the extent of their expression in a given patient’s PMaE or TME is likely to occur along a spectrum from fully Th1 to fully Th2 dominant and is likely to be determined by the combined influence of genomic and exposomic factors such as those described in Section 3. Some of these factors, such as genomic influences, would be expected to be stable. Others, like the patient’s metabolic status or cytokine milieu, would be expected to be changeable and could thus be amenable to clinical targeting.

First and perhaps most important, Th1 cells make IFNγ. IFNγ–STAT1 (signal transducer and activator of transcription 1) signaling induces SOCS1 (suppressor of cytokine signaling 1), inhibiting IL-4–STAT6 signals. STAT6 is a master regulator of Th2 cell expression and production of the key Th2 effector cytokine IL-4. Thus, inhibition of IL-4-STAT6 inhibits Th2 cell function (83). It is noteworthy that IL-4 acts as both a Th2 differentiation cytokine, inducing naïve CD4+ T cells to become Th2 cells, and also acts as effector cytokine, generated by Th2 cells to carry out Th2 effector functions like increasing mast cell numbers and function and also providing T cell help for B cell function (41). Th2 cells make IL-4 that activates STAT6, creating an autocrine loop. IL-4–STAT6 signaling also induces SOCS1 and SOCS3 expression in Th1 cells. SOCS1 inhibits IFNγ–STAT1 signaling (83). The second mechanism by which the Th1/Th2 reciprocal inhibition takes place is via competition for polarization of naïve T cells. Cytokines and other factors that drive a naïve CD4+ T cell to mature as a Th2 cell prevent the same naïve CD4+ T cell from becoming a Th1 cell and activating other cells of the Th1 system, or vice versa. This constitutes a simple opportunity cost (86). Third, IFNγ and IL-4 function as both differentiation cytokines (driving polarization of naïve CD4+ T cells to Th1 or Th2 cells respectively) and as effector cytokines (driving downstream functions that are part of the Th1 or Th2 immunological program) (87–89). Thus, Th2 cell production of IL-4 drives other naïve CD4+ T cells to become Th2 cells and also reinforces polarization commitment of existing Th2 cells, to sustain their Th2 polarization (41).

The same reciprocal inhibition described for Th1 vs. Th2 cells also applies to the epigenetic expression mechanisms governing phenotypic expression of M1 vs. M2 macrophages (90–93) and innate lymphoid cells type 1 vs. type 2 (68).

The effect of this reciprocal inhibition in the current discussion is that the patient whose immune system is characterized by upregulation of the Th2 system is likely to have a diminished Th1 response, so that the patient’s Th1 system may not be able to effectively respond to their cancer. This would be likely to be associated with poorer prognosis. The practical effects of Th2 inhibition of the Th1 cell system that would be concerning in the context of cancer include a) prevention of Th1-mediated anti-cancer surveillance, b) prevention of Th1 cytokine-mediated autocrine promotion of further naïve CD4+ T cell commitment to Th1 polarization and reinforcement of existing Th1 polarization commitment, and c) prevention of Th1 cell production of the Th1 effector cytokine IFNγ (41, 83, 94, 95).

It is noteworthy that all of the cells and cytokines of the immune system play a role in normal immune function. This raises the concern that, if the Th2 system is viewed as a candidate for treatment aimed at complete suppression of function, there may be a point beyond which such suppression may yield poor clinical outcomes. For example, IL-4 is a key Th2 system cytokine. In a mouse model, an IL-4 fusion protein (Fc-IL-4) has been shown to revitalize terminally exhausted CD8+ T cells, improving their tumor-killing functionality (96). This may imply that immunological factors like IL-4 that play a tumor promoting role when present at high levels may nonetheless be essential at lower levels for intact immune function.

#### 2.2.2 The role of macrophages in the TME

Tumor associated macrophages (TAMs) exhibit substantial plasticity and can alter their phenotypic expression along a continuum of polarization states, in response to stimuli in their environment (90, 91, 97). This makes them potentially useful treatment targets. TAMs have direct impacts on the TME that are tumor killing or tumor supportive, depending upon their phenotypic expression. TAM phenotype is also strongly influenced by T cell phenotypic expression (98) following the pattern of the reciprocal inhibition between Th1 and Th2 immunity, with M1 TAMs associated with an anti-tumor TME (10) and M2 TAMs associated with a tumor permissive TME (99).

TAM phenotypic expression is determined by signals from cytokines, prostaglandins, hypoxia, and metabolic factors and their related substrates and byproducts (10, 100–102). The M2-promoting influence of hypoxia has been reviewed in (103). Other studies have focused on the time course of tumor development, emphasizing factors like hypoxia in the evolving TME that, with time, influence macrophage phenotype toward M2. This topic has been reviewed in (76).

The phenotypic state of a given macrophage is not persistently determined, suggesting that macrophages are potentially a particularly useful target for treatments aimed at influencing the mix of signals affecting macrophage phenotype. Macrophages, including TAMs, oscillate between phenotypic states, including multiple phenotypic states beyond the originally conceived M1/M2 binary paradigm. The continuum of macrophage phenotypic expression along a spectrum between classically described M1 and M2 states has been reviewed in (76).

Some authors have suggested that the M1/M2 macrophage polarization model should be replaced with a model that more fully describes the complexity of macrophage phenotype along an M1-M2 continuum (92, 93, 102, 104). It is also worth noting that four types of M2 macrophages (M2a, M2b, M2c, M2d) have been described (10).

Nonetheless, in the context of cancer, it is still particularly useful to understand macrophage polarization as occurring along a continuum between M1 and M2 (90, 91). Overall, to the extent that a macrophage expresses the M1 phenotype, it participates in cytokine signaling programs and related factors that drive a Th1 system phenotypic shift and associated downstream responses. Likewise, to the extent that a macrophage expresses the M2 phenotype, it will drive Th2 system differentiation and downstream related responses. It is thus appropriate to understand descriptions of therapeutic influences targeting macrophage polarization as impacting phenotypic expression of the macrophage along this continuum, as well as influencing the Th1 or Th2 system of which the M1 or M2 macrophage respectively are a part.

TAM depletion and TAM re-education or reprogramming are two anti-tumor treatment strategies (105–110) that reflect the utility of either eliminating or reprogramming TAMs whose phenotype supports tumor instantiation and progression.

TAMs can account for 40 to 50% of the cells in some tumor types (100, 111). TAMs include both embryonic-derived tissue-resident macrophages (TRMs) and monocyte-derived macrophages (MdMs). Both TRMs and MdMs are significantly influenced by the TME (112–114), and both macrophage types contribute to prognosis for tumor growth and metastasis, so the influence is bidirectional.

##### 2.2.2.1 Macrophage TCA breaks and other influences

Macrophage phenotypic expression (including TAM phenotypic expression) is strongly influenced by the metabolic processes of the macrophage. M2 macrophages derive energy from the intact TCA cycle and oxidative phosphorylation. M1 macrophages are characterized by TCA cycle breaks and derive energy primarily from glycolysis (9). Macrophages are subject to reprogramming toward glycolysis, driving M1 polarization, in response to TLR4 stimulation by LPS, inflammatory cytokines or other factors (34).

A higher incidence of TCA breaks can also be driven by dysregulation of whole-body metabolic processes (35–37). Thus, metabolic dysregulation affecting the whole body can contribute to the incidence of TCA breaks in both parenchymal tissue and tissue resident macrophages.

In addition to influencing macrophage phenotypic expression toward the M1 phenotype, TCA breaks at succinate dehydrogenase (SDH), fumarate hydratase (FH), and isocitrate dehydrogenase (IDH), and IDH mutations also generate increased levels of oncometabolites succinate, fumarate, citrate, and D-2-hydroxglutarate respectively. These oncometabolites drive oncogenic transformation in the cells in which they arise (11, 12).

When succinate, fumarate, and citrate from these same TCA breaks drive TAMs to polarize to the M1 phenotype (11, 12), the M1 macrophages drive both direct anti-tumor effects and upregulation of the Th1 system.

Thus, to the extent to which metabolic dysregulation or other influences on the TCA yield TCA breaks, the resulting TCA break-associated metabolites play a dual role as oncometabolites that affect parenchymal tissues and as promoters of M1 phenotypic shifts that upregulate the Th1 cell system. M1/Th1 system upregulation is associated with inhibition of tumor development and better anti-cancer immune surveillance. The authors hypothesize that, in the immunologically normal patient, the oncogenic and M1-promoting effects of TCA breaks oppose each other, increasing the likelihood that the oncogenic influence will be effectively opposed by the normal immune surveillance provided by the M1/Th1 system.

Intact TCA function, in the absence of specific breaks, yields M2 polarization of macrophages (85) which inhibits macrophages from shifting to the M1 phenotype (29). Likewise, intact TCA function does not generate oncometabolites.

Consistent with the well-established literature, and central to the authors’ hypothesis, is the fact that, when TCA breaks occur in the Th2 dominant patient, the TAM shift to the M1 phenotype and consequent upregulation of the Th1 system will be diminished in proportion to the inhibition (32, 33, 49, 50, 63), but the metabolites created by the TCA breaks will nonetheless be oncogenic. To the extent that the M1/Th1 system activation is inhibited, the anti-cancer immune response is likely to be muted, yielding a greater likelihood that the oncometabolites will drive tumor development and progression. The strength of each of these effects will manifest differently in different patients, according to the genetic and epigenetic individuality of the metabolic, immunological, and other variables that influence each patient’s equation.

##### 2.2.2.2 M1 macrophages drive Th1 system activation

M1 macrophages (also called classically activated macrophages) are activated by IFNγ and microbial products such as LPS. M1 macrophages produce interleukin-12 (IL-12), which influences naïve CD4+ T cells to become Th1 cells (41, 90, 115), reinforces the polarization commitment to Th1 (115, 116), and increases the number and activity of natural killer cells (117). Th1 cells and NK cells produce interferon gamma (IFNγ) which stimulates the activity of CD8+ cytotoxic T lymphocytes (CTLs) and also increases the number and activity of M1 macrophages. This reinforcing activation loop sustains the numbers and activation of the cells of the Th1 system (30–33). The IFNγ generated through this process also contributes to Th1 polarization of naïve CD4+ T cells (118). In addition, IL-12 and IFNγ drive recruitment, activation, and antiviral effector functions of CD8+ T cells (8, 119, 120). IL-12 stimulates CD8+ T cells to produce IFNγ (121), creating further loop reinforcement of the Th1 system. (**Figure 3**) This loop reinforcement has been reviewed by Arora, et al. (122).

**Figure 3:**
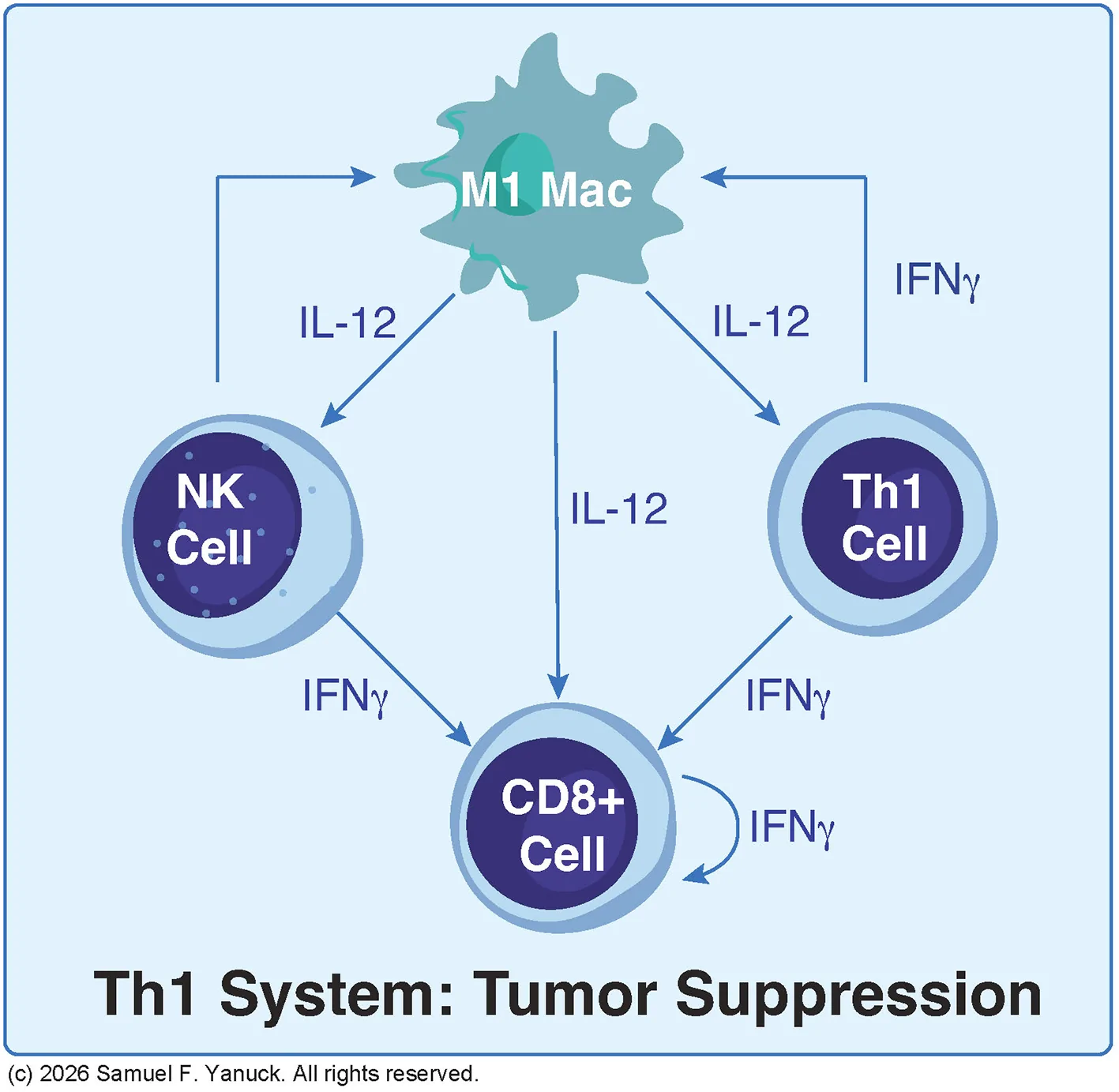
Cytokine mediated mutual activation of Th1 system cells. M1 macrophages produce IL-12 that drives numbers and activity of Th1 cells, NK cells, and CD8+ T cells. These cells in turn generate IFNγ that drives M1 macrophage activation, creating the activation loop that sustains activation of the Th1 system. IFN, interferon; IL, interleukin; mac, macrophage; NK, natural killer; Th, T helper cell.

CD8+ T cells also activate tumor necrosis factor/inducible nitric oxide synthase (TNF/iNOS)-producing dendritic cells (Tip-DCs). Tip-DCs prime naïve CD4+ T cells for proliferation and IFNγ production (123). M1 and M2 macrophages have been shown to generate cytokines that activate or inhibit NK cell functions respectively (124). The reciprocal inhibition between Th1 associated vs. Th2 associated signaling mechanisms within the macrophage that give rise to the M1 vs. M2 macrophage phenotypes have been reviewed by Chen, et al. (125) and by Kerneur et al. (126). Several authors have also used antibodies to target transmembrane proteins in M2-like TAMs, with encouraging early results in pre-clinical and early clinical settings (127–129).

Because M1 macrophages drive activation of the Th1 system, successful M1 macrophage polarization and activation of the Th1 system are crucial steps in mobilizing anti-cancer surveillance. Thus, intentional skewing of macrophages toward M1 polarization may be a useful clinical target.

It is noteworthy that, while IFNγ plays an important role in antitumor immunity, there are patients who are resistant to cancer immunotherapies that induce IFNγ production. IFNγ can also promote expression of genes associated with pro-tumorigenic effects, such as triggering of apoptosis in immune cells (130).

### 2.3 Tumor-generated signals that affect T cell polarization in the TME

In addition to the effects that CD4+ T cells of various polarizations have on each other, the tumor cells themselves also influence the immune cells in the TME (10, 131–135). Tumor cells secrete cytokines that promote factors favorable to tumor survival and growth. These include transforming growth factor β (TGF-β), interleukin (IL) 10 (IL-10), and IL-13, all of which promote Treg numbers and activity, driving immunological tolerance, as well as IL-4, IL-25, IL-33, and others that promote polarization of CD4+ T cells, macrophages, ILCs, and neutrophils to the Th2, M2, ILC2, and N2 polarization, respectively. The Tregs and Th2 system cells secrete cytokines that promote and reinforce tumor activity (tumor support). This constitutes a crosstalk between the tumor cells and the Tregs and Th2 system cells.

This crosstalk is relevant to the authors’ hypothesis in that the authors are proposing a patient macroenvironment assessment profile (PMAP) as a strategy for influencing the TME. This tumor-immune cell crosstalk influences the TME toward maintenance of the Th2/Treg dominance that drives poorer prognosis. Strategies aimed at modifying the TME need to be effective enough to overcome the influence of this crosstalk.

## 3 The patient macroenvironment

A key component of the authors’ hypothesis, supported by evidence from the literature, is that the immunological and metabolic status of the patient’s PMaE have a determinative influence on the TME and thus on patient prognosis (15, 136, 137). PMaE influence on T cell polarization status is of particular importance in this regard. The evidence cited here suggests that the influences between the PMaE and TME play a crucial role in determining whether the TME will suppress or support tumor growth. Whether this association represents a causal connection remains to be proven.

The subsections in this section discuss aspects of the PMaE, its influence on the TME, the impact of specific comorbidities and other factors on the PMaE and thus the TME, and barriers to identification of contributing factors.

### 3.1 Macroenvironment influence on T cell polarization

There are important, bidirectional influences between the TME and the patient’s overall immunological status, termed the patient macroenvironment (PMaE) or by some authors the tumor macroenvironment. These interactions and their implications for patient outcomes have been extensively reviewed (15, 16, 138, 139). Key components of this bidirectional influence include immunological, hypoxic, metabolic, pH, and mechanical factors (38); upregulation of Tregs and MDSCs, decreased diversity of T cell receptors (TCR), reduced production of IFNγ, and reduced NK cell cytotoxic potential (138); chronic inflammation, metabolic dysregulation, polymorphic microbiomes, aging, neuro-endocrine axis, stress responses, and metastatic colonization (15); metabolism, nutritional status, sarcopenia, systemic inflammation, organ dysfunction, and microbiome (16), stroma-tumor cross-talk (140) and the impact of methylation (141, 142). Some authors place particular emphasis on the impact of metabolism on the TME (143) and PMaE (137).

Crucially, factors consistent with promotion of a Th2 dominant PMaE will downregulate M1 macrophage and Th1 system activation. The upregulation of Tregs and MDSCs and reduction in Th1 system activity (IFNγ signaling and NK cell cytotoxicity) are consistent with the description of the protumor TME. Whole blood gene expression associated with CD8+ T cells and Th1 cells is reduced in patients with breast cancer compared with controls and mice with tumors experience significant changes in the composition and function of the immune macroenvironment, including changes in susceptibility to bacterial or viral infection, consistent with loss of Th1 system activation (144).

Naïve T cells transform into mature T cell phenotypes in response to differentiation cytokines, generated by antigen presenting cells and other cells in the lymph node and tissue environments. In 1986, Mossman et al. described the first polarization patterns, which involved the Th1/Th2 binary (145). Since then, at least five more T cell polarization types have been identified, depending upon whether CD28null T cell are classified as a separate phenotype (146–148). Despite the identification of these other T cell phenotypes, there remains a reciprocal inhibition between cells and cytokines of the Th1 and Th2 systems, so that elevation in one suppresses the other, controlled by a complex set of cytokines, receptors, gene expression patterns, and other mechanisms that regulate, among other factors, the balance between Th1 and Th2 cells (41, 83), M1 and M2 macrophages (29, 90, 149) and ILC1 and ILC2 cells (150–152).

### 3.2 Comorbidities associated with Th2 dominance

Assessing the extent of Th2 dominance in the PMaE includes an assessment of comorbidities known to involve Th2 immunity. Thus, it is appropriate to examine the research basis for connections between specific diseases, their underlying Th2 dominance, and their association with cancer risk.

Some disease processes are associated with upregulated expression of Th2 system cells. Asthma, specifically T2-high asthma, is a Th2 dominance–based disease (153), though there are nonatopic and T2-low forms, the former now understood to involve Th17 polarization and generation of IL-17 by Th17 cells, driving proliferation of neutrophilic inflammation rather than eosinophilic inflammation (154). In a large cohort study, patients with asthma had nearly triple the incidence of cancer, led by breast, colon, lung, and prostate cancer (13). In a much larger multisite study, asthma was again associated with greater cancer risk, with 15 of 17 sites showing a greater risk in their subject cohorts, and with hazard ratios that ranged from 1.09 to 1.94 depending upon the site (14).

There are associations of Th2 disease with better cancer outcomes that may be cancer specific, providing protection in some (155), but not in others (156).

### 3.3 Th2 dominance in other diseases

Other named disorders are also known to involve Th2 dominance. These include environmental allergy (157, 158), atopic dermatitis (159, 160), most chronic rhinosinusitis (161, 162), eosinophilic gastrointestinal disorders (163–165), and food allergy (166, 167).

Gastrointestinal and pulmonary epithelia generate thymic stromal lymphopoietin (TSLP), IL-33, and IL-25 alarmins, which are a subgroup of damage associated molecular patterns (DAMPs). These cytokines drive naïve T cells to mature as Th2 cells and drive innate lymphoid cells to mature as innate lymphoid cells type 2 (ILC2’s). Thus, any GI disorder or pulmonary disorder that involves epithelial cell perturbations resulting in inflammation will drive the patient toward Th2 dominance (168–170). And because DAMPs are themselves pro-inflammatory, they tend to perpetuate inflammation in the spaces in which they are generated, reinforcing the biology that gives rise to their generation. Similar immunological patterns involve the sinuses and skin.

Once innate lymphoid cells polarize into ILC2’s and naïve T cells polarize into Th2 cells, they make the effector cytokines IL-4, IL-5, and IL-13 that carry out their immunological program. Crucially, IL-4 drives mast cell proliferation and degranulation, and B cell IgE production. IL-4 also functions as a differentiation cytokine, driving Th2 polarization of other naïve CD4+ T cells and reinforcing Th2 polarization of existing Th2 cells. This makes IL-4 an autocrine loop activator of the Th2 system. Other Th2 cytokines also drive important processes. IL-5 drives eosinophil proliferation and activation. IL-9 drives basophil activation and mast cell hyperplasia. IL-13 drives mucus production and basement membrane thickening (17, 26, 168–170). ILC2-derived IL-13 also drives proliferation and activation of MDSCs (171). MDSC proliferation and activation is also driven by TGF-β, a key driver of tolerogenic signaling in the TME that, in high levels, activates cancer-associated fibroblasts (CAFs), induces epithelial-mesenchymal transition (EMT), and hinders immune cell function (172, 173). MDSCs produce TGF-β (174, 175), forming a reinforcing loop that contributes to maintenance of the tolerogenic program that reinforces the problematic TME.

TSLP and IL-33, alarmins generated by stimulated epithelial cells, drive mast cell proliferation and degranulation, yielding histamine production, in an IgE-independent manner (176). Mast cell degranulation releases proteases, histamine, and the inflammatory cytokines TNFα and IL-1β, which together increase epithelial barrier permeability (177). TNFα and IL-1β drive further epithelial cell-induced inflammation, stimulating further TSLP, IL-33, and IL-25 generation, reinforcing the original epithelial cell-induced inflammation. Mucosal mast cell numbers are increased in irritable bowel syndrome and functional diarrhea (17). Mast cell degranulation also releases mast cell mitochondrial DNA (mtDNA) fragments, known to drive inflammation in Crohn disease and ulcerative colitis (178). These mtDNA’s stimulate mast cells in an autocrine and paracrine fashion (179). As with TNFα and IL-1β, mtDNA’s released by mast cell degranulation would thus be expected to reinforce epithelial release of TSLP, IL-33 and IL-25 that drive Th2 dominance.

### 3.4 Barriers to identification of Th2 dominance in the PMaE

The development of an accurate PMAP depends upon identification of patient immunological status that goes beyond an inventory of existing formal diagnoses. Many factors that influence immunological status toward Th2 dominance are present in the patient’s biology but are not reflected in specific diagnosis codes, either because the phenotypic expression is partial (so the patient does not meet criteria for the diagnosis) or because they are clinically relevant (like the impact of stress on immune function) but are not diagnostically categorized clinical entities. Because of these considerations, it is important to assess the state of the literature for limitations that may present challenges to the development of an effective PMAP.

The task of characterizing patient T cell polarization and the identification of patients with a Th2 dominant phenotypic expression is complicated by variations in T cell polarization that are not associated with atopic disease. These patients may not carry diagnoses that identify them as Th2 dominant. This may yield an incomplete identification of Th2 dominant patients in retrospective studies that depend upon database searches for diagnostic codes. Biological influence toward Th2 dominance in these patients may be induced by the effects of exposomic effects described below.

Meta-analyses and retrospective studies that rely upon diagnosis code data from existing databases may incorrectly identify a patient as Th2 dominant if a diagnosis of asthma or other atopic disease persists in their medical record, despite resolution of disease or a shift to a milder phenotypic expression, such that they would no longer meet criteria for the diagnosis their medical record describes. Other challenges to accuracy of patient characterization include case complexity and changes in severity of key immunologically relevant comorbidities over time. The impact of the Th2-promoting exposomic influences described below are not accounted for, since they typically don’t get represented in the patient’s chart unless they are themselves of sufficient severity to warrant a separate diagnosis. Even then, retrospective studies of cancer patients’ immunological status typically don’t categorize patients in a way that includes consideration of these factors. Changes in intensity of the patient’s primary Th2-dominance-based comorbidity also complicate the task of accurately accomplishing group characterization for subject records in retrospective studies or, by extension, meta-analyses.

Accurate identification of patient immunological status is further diminished by combining diagnoses like atopic asthma with non-asthmatic atopic conditions like hay fever. Lastly, the immune system is not a single entity that can be described as activated or diminished in its function. Cells and cytokines can influence each other in ways that can signal for activation of function, proliferation, inhibition of function, etc. So, terms like “immune activation” have no specific meaning. Activation of the Th2 system supports tumor biology. Activation of the Th1 system inhibits the Th2 system and supports anti-cancer surveillance.

### 3.5 The role of obesity as a patient macroenvironment factor

According to Shen, et al., “overweight and obesity account for approximately 10% of new cancer diagnoses annually in the US and up to 50% of certain cancers such as endometrial and hepatobiliary cancer.” (reviewed in 180). Body mass index is positively associated with risk for 19 cancer types (reviewed in 181).

There are several examples of the link between cancer immunology and obesity. In cancer, obesity-associated metabolic and related factors create immune suppression, including suppression of CD8+ T cells and NK cells, and upregulation of immune suppressive MDSCs (reviewed in 182–184). Jimenez-Cortegana et al. (185) and Sanchez-Pino (186) both report an association between obesity-related MDSCs and increased tumor development, dissemination, progression, and poor clinical outcomes. Evidence also shows that MDSCs promote the development of tumor associated Tregs that promote effector T cell anergy (187).

Obesity induced by a high fat diet (HFD) impairs CD8+ T cell functionality. In the context of HFD-induced obesity, tumor cells increase fatty acid uptake, creating fatty acid scarcity that impairs CD8+ T cell function and diminishes anti-tumor immunity (188).

Scarcity of fatty acids and other nutrients is also driven by metabolic shifts in the TME which deprive effector T cells of nutrients and energy substrates. Tregs are able to adapt to the lipid-rich tumor microenvironment by relying upon fatty acid oxidation, conferring a survival advantage and supporting an immune suppressive TME (189).

Obese individuals have been shown to have fewer circulating NK cells. NK cells from obese individuals have been shown to produce less granzyme and perforin, kill fewer tumor cells, and produce significantly less IFNγ, when compared with NK cells from lean individuals (190).

In obesity, activation of the Wnt/β-catenin signaling pathway promotes an M2 phenotypic shift in TAMs (reviewed in 191). Obesity is also associated with upregulation of angiogenesis, which can drive cancer cell survival, proliferation, and metastasis (192, and reviewed in 193, 194).

Chronic low-grade systemic inflammation, common in obese patients, elevates pro-inflammatory cytokines, recruiting Th1 cells and CD8+ cytotoxic T lymphocytes, which subsequently suffer functional exhaustion. The T cells of obese patients demonstrate an increased expression of the programmed cell death protein-1 (PD-1), a cell surface receptor that acts as an “off switch” for the T cell. When tumor PD-L1 (the ligand for PD-1) meets T cell PD-1, it turns off the T cell so it cannot destroy the cancer, thereby promoting tumor survival. Lastly, continuous interaction of these T cells with tumor cells yields T cell exhaustion, favoring immune evasion and tumor growth (reviewed in 195).

## 4 Exposomic influences

Alterations in patient biology that are not disease specific are nonetheless known to shift the PMaE toward Th2 dominance. There is a substantial body of research documenting the Th2-promoting influence on the PMaE for each of the exposomic elements described here. The smallest body of evidence is for the impact of sleep on T cell phenotypic expression. The largest body of evidence supports the role of stress as shifting T cell phenotypic expression to Th2.

As already stated, epidemiological studies describing a strong association between Th2-dominant diseases like T2 high asthma and significantly higher incidence of common cancers suggests that the Th2 dominant PMaE influences the TME toward Th2 dominance, explaining the association between atopic diseases like T2 high asthma and increased cancer incidence. Whether this association represents a causal connection remains to be shown.

Each of the elements described here has the potential to contribute to the total equation of T cell polarization in a given patient’s PMaE. The extent of a patient’s susceptibility to each of these influences, based on genetic, epigenetic, immunological, metabolomic, or other factors is a matter for future research, as is the relative strength of each of these influences in comparison with the others in the overall population.

It is worth noting that studies of the association of “allergic diseases” with cancer do not typically control for Th2-promoting elements, unless the patient carries a specific diagnosis related to the involved element(s).

The pulmonary system can be exposed to instigators of Th2 response, such as pollen, dust mites, other airborne allergens, diesel exhaust particles, chemicals, and air pollutants (26, 196). Immune response to wood smoke inhalant irritation measured in upper airways (nasally) has been noted to be sex specific (197) and in upper airways, associated with increased eosinophils in sputum (198). T cell function and polarization toward Th2 with chronic particulate matter exposure has been reviewed by Ma (199). The exacerbating effects of environmental pollutants on allergic (Th2 dominant) disease has been reviewed by Lopez-Retana et al. (200).

Intestinal dysbiosis (17) or subclinical gastrointestinal inflammation (201–204), can occur outside the context of overt disease manifestation, driving upregulation of IL-33, TSLP, and IL-25, as described above. Dysbiosis of the gastrointestinal microbiome is associated with both asthma (205) and allergy (206), though the mechanisms of these associations have complexity beyond the issue of Th2 dominance. Intestinal dysbiosis has also been associated with colon cancer development (207–210). Some pathogens that can colonize the intestine, including Aspergillus fumigatus (211, 2124), Candida albicans (213), and Staphylococcus aureus (214), employ evasion strategies that shift the host into a Th2 response, with the loss of the Th1 system response yielding an environment conducive to pathogen survival.

The Th2-polarizing effects of epithelial barrier exposure to environmental allergens, air pollutants, microplastics, nanoparticles, bacteria, viruses, fungi, hygiene products, processed foods, and other exposomic elements have been reviewed in (215).

Th2-promoting exposomic effects of elevated cortisol and/or norepinephrine from stress (18–23) have also been described. Stress chemistry, in the form of cortisol and norepinephrine, drives a shift in the patient macroenvironment into Th2 dominance (18–20, 24). Stressed mice are more likely to develop skin cancer (216). Th1 and NK cell apoptosis is induced by upregulation of cortisol and norepinephrine in traumatic brain injury, spinal cord injury, and stroke, as described by Meisel et al. Patients with these injuries have significantly greater incidence of ventilator-associated pneumonia than other patients (217). The literature on the immunological impact of stress, including Th1-inhibitory and Th2-promoting effects, has been reviewed in a meta-analysis covering 30 years of research, by Segerstrom et al. (218).

Further complicating patient characterization is the fact that a patient whose atopic disease is dormant or well controlled because of the use of corticosteroids may have their underlying Th2 dominance reinforced by these medications (18).

Older-age-associated changes in the immune response that contribute to a shift toward a cancer-permissive TME have been reviewed by Fane and Weeraratna (219) and include increases in M2 macrophages and N2 neutrophils, as well as immunosenescence of effector T cells, NK cells, macrophages, and dendritic cells. Older age is also associated with a decreased TCR repertoire (220). Research on T cell immunosenescence has been systematically reviewed by Rodriguez et al. (221).

The Th2-promoting effect of sleep dysregulation has also been described (24, 25), though the literature on this topic is not as abundant as for the other exposomic factors described here. Irwin (24) has reviewed the relationship between failure of sleep efficiency and diminished Th1 response, with a range of factors, including chronic stress and mild persistent inflammation, capable of inducing a failure of sleep efficiency, leading to increased inflammation, promotion of Th2, and failure of Th1-mediated antiviral effects.

Exposure to endocrine-disrupting plastic additives and surfactants is associated with low glutathione levels, suppression of Th1 cell development, and enhanced Th2 cell development (222–225). This literature has recently been reviewed (226, 227).

Each patient’s genome will influence the likelihood of phenotypic expression of any of the above factors. This topic, while of substantial importance, is beyond the scope of this discussion.

In order to design an effective PMAP, factors like those described above and their immunological implications need to be evaluated for their impact on patient immunological status.

Many exposomic elements, including some that have only appeared in modern times (air and water pollution, microplastics and other toxicants, other novel molecules, etc.) drive the PMaE toward Th2 dominance. The fact that so many commonly encountered individual exposomic elements influence the PMaE toward Th2 dominance suggests that there is likely to be a net effect on the PMaE that favors Th2 dominance in the population. This raises concerns that mirror the trend of increased incidence of certain cancers in the under 50 cohort (228).

## 5 TCA breaks and mutations

TCA breaks and mutations generate oncometabolites that are tumor promoting. They also drive macrophages to the M1 phenotype. The authors hypothesize that this dual pro-and-anti carcinogenic effect provides the setting in which inhibition of Th1 system activation in the Th2 dominant patient can potentiate tumor development and progression.

Macrophage polarization toward glycolysis and the M1 phenotype from TCA breaks occurs in response to TLR4 stimulation by LPS, inflammatory cytokines or other factors (34). As a separate influence, a higher incidence of TCA breaks can also be driven by dysregulation of whole-body metabolic processes (35–37). Thus, metabolic dysregulation affecting the whole body can contribute to the incidence of TCA breaks in both parenchymal tissue and tissue resident macrophages.

### 5.1 Oncogenic and Macrophage polarizing effects of TCA breaks and mutations

Central to the authors’ hypothesis is the body of evidence showing that breaks and mutations in TCA cycle enzymes yield the dual effects of oncometabolite production and M1 macrophage phenotypic expression. In patients whose PMaE is Th2 dominant, IL-4 and other cytokines generated in greater abundance inhibit the patient’s ability to mount an effective Th1 response. Cancer cells are not eliminated, tumor growth follows, and the patient’s TME is Th2 dominant, yielding poorer prognosis.

Mutational and non-mutational impairments in three TCA cycle enzymes: succinate dehydrogenase (SDH), fumarate hydratase (FH), and isocitrate dehydrogenase (IDH), have recently been identified as drivers of oncogenic transformation, as reviewed by Laurenti and Tennant (27) and by Eniafe and Jiang (28). TCA breaks at IDH, SDH, and FH yield accumulation of the oncometabolites citrate, succinate, and fumarate, respectively. IDH mutational changes yield conversion of existing alpha ketoglutarate to D-2-hydroxyglutarate (D-2-HG), a process associated with angiogenesis (229, 230).

Among several mechanisms, D-2-HG promotes epigenetic regulation of oncogenic gene expression, decreased activity and proliferation of T cells, T cell differentiation skewing toward the Treg phenotype, and increased oxidative stress (28, 231). Accumulation of succinate and fumarate in tumors promotes pseudohypoxia by mimicking hypoxia through inhibition of prolyl hydroxylases. This stabilizes hypoxia-inducible factor (HIF) 1α, promoting expression of HIF1α target genes associated with glycolysis, angiogenesis, and epithelial-mesenchymal transition. Succinate secretion by tumor cells also promotes macrophage polarization in TAMs (28). Citrate, at low concentration, appears to support oncogenic mechanisms like fatty acid synthesis and histone acetylation in tumors. At high levels, it has been described as cytotoxic (232, 233).

The broader tumor-promoting implications of these defects have been reviewed by Guo et al. (234). The implications include remodeling of tumor cell metabolism to provide building blocks for tumor cell biosynthesis and proliferation, cancer cell utilization of nutrients that are therefore unavailable to normal immune cells, upregulation of reactive oxygen species, and production of cancer-associated metabolic intermediates, including succinate and D-2-HG (27).

### 5.2 Loss of SDH and IDH shifts macrophages to M1 phenotype

M1 macrophages rely upon glycolysis for TCA cycle ATP generation, whereas M2 macrophages rely predominantly upon the slower but higher ATP generating process of oxidative phosphorylation. This contrast, and the associated TCA breaks at SDH and IDH that characterize the metabolism of the M1 macrophage phenotype, have been described by O’Neill et al. (29, 34, 235, 236).

The SDH enzyme break leads to accumulation of succinate that is necessary for macrophage cytokine function. Succinate accumulation also leads to mitochondrial accumulation of metabolites necessary for macrophage immune functions. The IDH break leads to accumulation of isocitrate and citrate, which are used to generate fatty acids for membrane biogenesis, as well as prostaglandins and nitric oxide. Citrate is also converted to itaconate, which has antimicrobial functions (29). Itaconate is also required for the anti-inflammatory function of cortisol (237).

Likewise, TCA breaks at FH yield a shift in macrophages to glycolytic processes associated with the M1 phenotype (238, 239). **Figure 4** describes the mix of oncometabolite generation and M1 phenotypic influence of TCA breaks and mutations (**Figure 4**).

**Figure 4:**
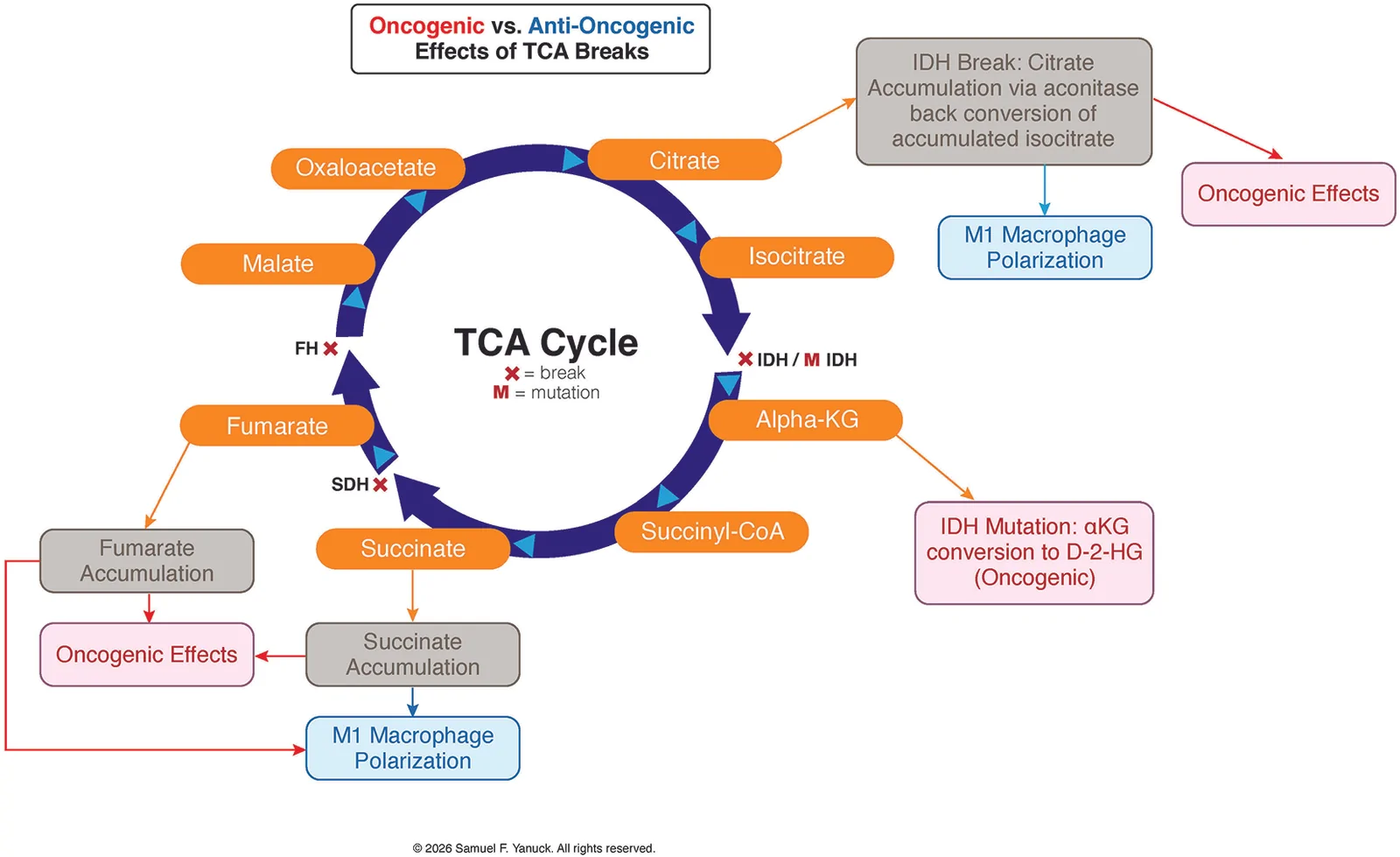
Oncogenic and M1 macrophage promoting effects of TCA breaks and IDH mutations. TCA breaks and mutations drive oncometabolite formation associated with malignant cellular transformation. The same TCA breaks and mutations drive activation of glycolysis in macrophages that drives their phenotypic shift to the M1 macrophage phenotype, activating the Th1 system. CoA, coenzyme A; FH, fumarate hydratase; IDH, isocitrate dehydrogenase; KG, ketoglutarate; mac, macrophage; SDH, succinate dehydrogenase.

Thus, the same TCA breaks at SDH, IDH, and FH that promote oncogenesis in the tissues in which they arise also transform macrophages to the M1 phenotype necessary to promote Th1 system activation associated with anti-cancer immune surveillance in those tissues. Moreover, the Th1 system activation yields an increase in IFNγ and favorable downstream effects like IFNγ-mediated conversion of 25OHD3 to 1,25(OH)2D3 required for macrophage phagocytosis and for reduction of inflammation that favorably affect the PMaE and TME. A key component of the authors’ hypothesis is that, in individuals without cancer, the oncogenic influence of TCA breaks is counterbalanced by the shift in macrophage phenotype to M1, driving the resulting upregulation of the Th1 system and its associated anti-cancer surveillance. In the normal patient, the Th1 system cells are more likely to clear the oncogenically transformed cells, so that the patient doesn’t develop cancerous tumors. However, in the Th2 dominant patient, the Th2-mediated inhibition of the Th1 system prevents upregulation of the Th1 system, making it more likely that the oncogenic transformation will succeed in driving tumor formation.

## 6 The integrated picture

**Figure 5** shows the components and their interconnections that comprise the integrated picture described by the authors’ hypothesis, including: 1) the inhibitory or reinforcing effects on tumor biology from Th1 immunity and Th2/Treg immunity respectively, 2) patient-specific Th2 promoters and comorbidities in the PMaE and their Th2 promoting influence on the TME, 3) the effects of TCA breaks and mutations driving both oncometabolite formation that is tumor promoting and conversely M1 phenotypic expression of macrophages that activates the Th1 system, and 4) inhibition of the Th1 system by the tumor, the Th2 system, and the Regulatory system (**Figure 5**).

**Figure 5:**
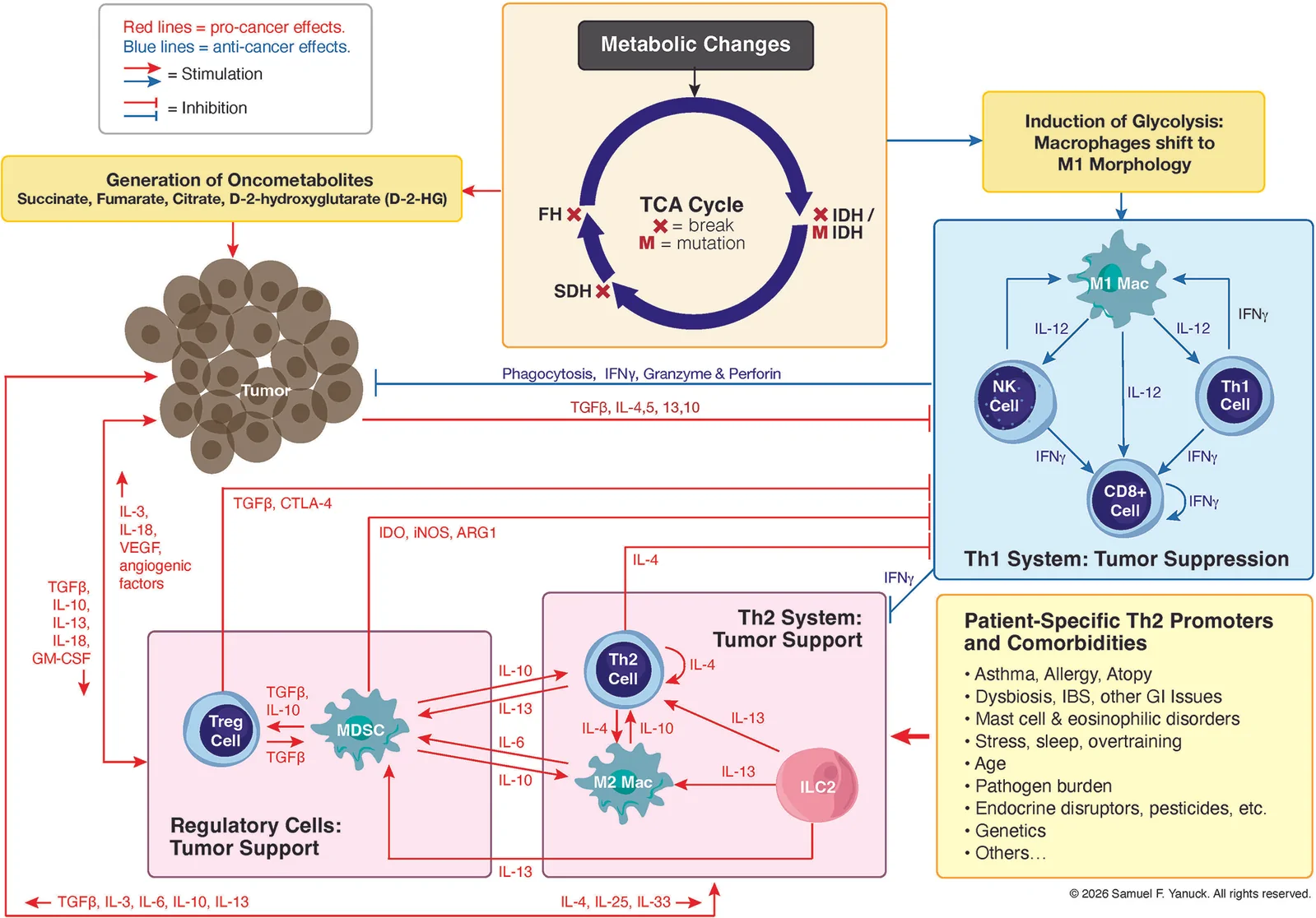
Connections between TCA breaks and IDH mutations, tumor microenvironment, and patient macroenvironment. In the mitochondria, TCA breaks at SDH, IDH, and FH, and IDH mutations, yield accumulation of succinate, citrate, D-2-HG, and fumarate, which drive tumorigenesis. The TCA breaks also drive a shift in macrophage metabolism toward glycolysis that is associated with a shift in macrophages to the M1 phenotype. M1 macrophages drive activation of the Th1 system (blue box), which inhibits tumor development. The tumor itself promotes anergy of both CD4+ (all T cells shown) and CD8+ T cells (in blue box). The Th1 system is inhibited by the Th2 and regulatory cell systems (pink boxes). The Th1 system inhibits the Th2 system. The tumor, regulatory cells, and Th2 system reinforce each other through multiple mechanisms. Patient-specific Th2 promoters and comorbidities (yellow box) are associated with greater Th2 system expression, reflecting the influence of the PMaE on the TME. ARG1, arginase 1; CRS, chronic rhinosinusitis; CTLA, cytotoxic T-lymphocyte associated protein, EDC, endocrine-disrupting chemical; FD, functional diarrhea; FH, fumarate hydratase; GI, gastrointestinal; GM-CSF, granulocyte-macrophage colony-stimulating factor; IBS, irritable bowel syndrome; IDH, isocitrate dehydrogenase; IDO, indoleamine 2,3-dioxygenase; IFN, interferon; IL, interleukin; ILC2, innate lymphoid cell type 2; iNOS, inducible nitric oxide synthase; mac, macrophage; MDSC, myeloid-derived suppressor cell; NK, natural killer; SDH, succinate dehydrogenase; TCA tricarboxylic acid; TGF, transforming growth factor; TRAP, traffic-related airborne pollution; Treg, regulatory T cell; VEGF vascular endothelial growth factor.

## 7 Discussion

TME’s with greater numbers of Th1 cells have been associated with improved outcomes in human cancer trials (42–47). TME’s with greater numbers of Th2 cells have been associated with poorer outcomes in human cancer trials (51–55). From an Implementation Science perspective, it is sensible to create a clinical approach that allows clinicians to identify and address, on a patient-specific basis, exposomic and other influences that skew the patient’s PMaE and TME toward a Th2 system phenotypic expression. The authors propose that, in both research and clinical settings, attention should be paid to the connections between the exposome, PMaE, TME, and outcomes, with the goal of creating a clinically applicable inventory of lab markers, questionnaires, and other forms of assessment that identify and target patient-specific elements in the exposome and PMaE that skew a patient toward expression of the Th2 phenotype. Identifying and addressing these elements has the potential to shift the PMaE and TME away from Th2 and toward Th1 phenotypic expression, and thus favorably influence patient outcome.

The authors propose four main consequences of this approach. First, systematically addressing the through-line from exposome to PMaE to TME could improve overall patient outcomes, by improving patient responses to their existing treatments.

Second, there is a value to identifying the specific exposomic targets that matter most for each patient. It is not realistic to ask every patient to engage in every possible lifestyle and other exposomic intervention that could improve outcomes, including changes in diet, lifestyle, nutrition, sleep, stress, exercise, relationships, work, environmental exposures, and a host of other factors. A quantified patient assessment would facilitate the clinician’s ability to choose targets with the potential for highest impact on patient outcome and ignore others. This would be expected to greatly improve patient compliance, by keeping to a realistic set of instructions that the patient has a better chance of following.

Third, identifying and eliminating problematic exposomic influences on the PMaE could improve the risk profile for patients in cancer remission and for patients who do not have cancer but are at high risk for developing cancer.

Fourth, depending upon the context, it may be useful to evaluate the PMaE and TME as part of the routine of characterizing study subjects in cancer research. Assessing CD4+ T cell and macrophage polarization patterns allows patients to be randomized into treatment and control groups in a way that ensures a roughly equal number of Th2 dominant patients in both groups. This prevents circumstances in which a viable treatment fails to show significance because there are substantially more Th2 dominant patients in the treatment group versus the control group. Said another way, if Th2 dominance is a potential modifying factor in assessing the efficacy of a therapeutic drug or other treatment, controlling for Th2 dominance in a research subject’s phenotypic profile is a reasonable consideration.

### 7.1 Profiling the patient macroenvironment

As described above, substantial evidence supports the impact of the PMaE on the TME, and the importance of the TME in patient outcomes. Multiple factors essential to patient biology such as stress, diet, obesity, and other exposomic influences discussed above, are known to impact the PMaE. Thus, there is a through-line connecting common factors in patient biology like stress, diet, obesity, and other exposomic influences, with their impact on the PMaE, connecting the PMaE with its impact on the TME, and connecting the TME with its impact on patient outcomes. On that basis, it is the authors’ view that future study should aim to develop and prospectively validate a suite of assessments that provides a quantified patient macroenvironment profile (PMAP). The authors hypothesize that a working PMAP can be developed that, upon cancer diagnosis, would provide information about targets in the PMaE that could be addressed, with the goal of influencing both PMaE and TME, in service of improving patient prognosis. The PMAP is proposed as a conceptual framework, rather than as an evidence-supported clinical profile, though the authors’ proposed goal would be to develop the PMAP so that it could function as a practical clinical tool in the ways described in this paper. The following discussion reflects the authors’ approach to starting the PMAP design process, with the goal of developing the PMAP into an evidence-based instrument that could be used in clinical practice, in support of better patient outcomes, in addition to and in support of the patient’s primary treatment methods, rather than instead of them.

#### 7.1.1 Development and validation of the PMAP

Several steps would be required to develop a working PMAP. First, a specific cancer would need to be selected for study. Prostate cancer (PCa) would be a useful candidate, as obtaining biopsy samples is straightforward and repeat biopsy is a routine practice. Biobanked tumor samples, blood and other samples could be obtained and analyzed using metabolomic, proteomic, and other platforms. These data could be combined with genomic information, details about tumor type, stage, and grade, comorbidities including those related to atopic/non-atopic status, and other data from patient EMRs. Appropriate analysis of these data would be a first step in modeling a PMAP.

Second, biospecimens from a prospective cohort of PCa patients with and without atopy could be obtained, with an N sufficient to power the study, for known metabolomic and exposomic markers (240–242) as well as immune, inflammatory, genetic, senescence and microbiome markers known to impact Th2 processes in the patient macroenvironment. A standard exposome evaluation instrument like the NIEHS Personalized Environment and Gene Study (PEGS) questionnaire could be used to collect exposomic data across the lifespan. All biomarker data could be coalesced into a final data set for computational modeling.

Data from the above process could then be analyzed using advanced computational correlational analyses to generate the final PMAP. AI algorithms and machine learning tools could be applied to the PMAP (ie. all readouts that separated based on +/- Th2 atopic disease) to optimize the PMAP.

#### 7.1.2 Clinical implementation

The PMAP implemented in clinical settings would consist of laboratory tests (blood, sputum, urine, etc.), patient history, dietary and related inventories, lifestyle and exposome questionnaires, and other evaluative tools. The purpose of evaluating patients with the PMAP is to provide information about factors discussed above that drive patient T cell polarization and other factors that influence the PMaE and thus the TME. This includes attention to diagnosed comorbidities with a known Th2 association such as Th2-high asthma, eczema, and other atopic disorders. And, because the patient’s biological status is influenced by a range of factors that may be present but for which no formal diagnosis has been made, due consideration must also be given to other common factors discussed above, such as stress, sleep, obesity, exercise, GI disorders such as SIBO or other microbiome disorders, pulmonary or sinus disorders, exposomic factors like work-place exposures, air pollution, or secondhand smoke, and other disorders which may be present, but for which the patient may not carry a formal diagnosis.

For comorbidities and other factors contributing to a patient’s PMAP, efforts should also be made to understand the extent of current illness severity versus, for example, viewing all patients carrying a Th2-high asthma diagnosis as having equal disease severity, which would incorrectly give them the same PMAP score-component for that factor.

PMAP evaluation would yield both a quantified index score and a set of biological targets that could be identified for treatment. Each of the factors evaluated should contribute to the total score according to frequency, duration, intensity, and relevance to immunological, metabolic, or other systems that impact the PMaE or TME. The PMAP index score should correlate with and predict known TME measures such as the Immunoscore (7, 80–82).

Crucially, factors identified in the patient’s PMAP should provide guidance about which of the evaluated factors ranked highest by contributing most to the patient’s score. Interventions could be aimed at these factors, with the goal of changing the patient’s PMaE and thus their TME. This would be done in support of the patient’s primary treatment options, not instead of them. Though stress, sleep, diet, obesity, exercise, and a host of other factors might contribute to the picture of cancer prognosis, it is nonetheless unrealistic to tell every patient that they need to focus on the whole list of possible changes that could improve their outcome. The PMAP information would provide quantified characterization of factors in the PMaE, scaling the importance of each factor relative to the others for a given patient, guiding decisions about which of the many lifestyle, exposome, and other factors are worthy of the patient’s attention, such that one patient might be advised to focus on sleep, stress, and sinus issues, where another might focus on digestion and dietary factors. This might substantially increase compliance, by focusing the patient on a smaller, more meaningful list of factors. It can also be used as a monitoring tool that contributes to and contextualizes assessment of factors contributing to disease progression, remission, treatment success or failure, and significant changes in lifestyle, all of which are important data points to include in a patient’s treatment regimen.

Focusing on these factors has the potential to make a meaningful contribution to optimization of patient outcomes across a broad spectrum of cancers, in addition to and in support of current approaches in oncology, with the goal of improving the outcomes provided by current treatment methods.

### 7.2 Risk screening

In patients at risk for developing cancer, and in cancer survivors, there is no tumor, so there is no TME. But there is always the whole biology of the patient, with immunological status, metabolic status, etc. This is the PMaE. As discussed above, the authors hypothesize that when TCA breaks and/or mutations occur, if the patient has a Th2 dominant PMaE, the upregulation of the M1/Th1 response in that patient’s tissues would be expected to be muted, so that the influence of oncogenic metabolites on the tissues in which they arise would not be countered by the anti-cancer surveillance of the patient’s M1/Th1 response. It is established that patients with asthma have more than double the risk of lung, breast, prostate, and colon cancer. The authors contend that the Th2 dominance of the PMaE in these patients is an important contributor to the greater risk for development of common cancers in these patients.

Thus, the authors contend that in high-risk patients, PMaE status is relevant to disease risk and PMaE evaluation and treatment presents a potentially important opportunity for adjunctive treatment in patients at higher cancer risk.

For cancer survivors, the same whole-patient biology of the PMaE that gave rise to cancer in the first place may still be present, still influencing the effects of TCA breaks and/or mutations, and still influencing T cell polarization. Even when cancer treatment is completely successful, the question of whole patient biology of the PMaE remains. The authors contend that it would be worthwhile to study whether cancer survivors whose PMaE is cancer-permissive are more likely to have recurrence of their cancer, compared to those for whom their current survivor status is accompanied by a more effective anti-cancer PMaE. For all the reasons discussed above, the authors suggest that using the PMAP to identify and address problematic factors in metabolism, T cell polarization, and other factors discussed above could reduce their risk of recurrence.

With the above considerations in mind, a fully developed PMAP could be part of cancer screening for patients at increased risk for developing cancer and for those at risk of cancer recurrence. The PMAP could be used to identify and modify factors that contribute to an unfavorable PMaE, as reflected by a high PMAP score. Factors contributing to the high PMAP score and thus an unfavorable PMaE could be addressed, with the goal of modifying the PMaE, the TME, and thus patient outcomes.

### 7.3 Impact on study randomization/stratification

Lastly, as the authors have discussed, the TME substantially impacts tumor biology and cancer outcomes. The authors suggest that, when randomizing patients for cancer trials, it may be important to characterize key immunological, metabolic, exposomic, and other PMaE factors that may influence the status of the patient’s TME. If this is not part of the randomization process, randomization of a study population might yield a treatment group with significantly more Th2 dominant patients than the control group. The Th2 dominance of these patients would be expected to mute their M1/Th1 response, preventing the patient’s own anti-cancer immune surveillance from influencing treatment outcome as much as would occur in the Th1 dominant patients. If there were enough Th2 dominant patients in the treatment group, this could represent enough of an influence to mask the effectiveness of the treatment being studied. This would make it more likely that the treatment being studied would fail to show efficacy. The difference between Th2 dominance and Th1 dominance might be especially important, for example, in the study of medications aimed at conserving T cells by preventing PD-1 ligation. It may be crucial in these studies to know if the patients being studied are Th2 dominant, since preservation of the T cells of Th2 dominant patients would not be expected to confer advantages in outcome. Thus, the authors contend that a PMAP or similar evaluation could be important to any cancer trial, in order to provide a more optimal randomization process and avoid avoidable trial failures.

### 7.4 Limitations, unresolved questions and future research

The conceptual framework and hypothesis presented here have limitations. For example, a valuable PMAP is one that includes important exposomic variables that contribute to the patient’s PMaE, ultimately revealing susceptibilities that can be identified as treatable targets for better overall outcomes. Therefore, optimal computational methods are needed to rigorously quantify exposomic variables. At present, these methods are not fully developed. A second limitation is that diseases that carry similar diagnoses or are coded broadly in medical records, such as “asthma” have important nuances that can differentially affect patients’ PMAPs, such as distinguishing T2 high versus T2 low asthma. Underappreciating these nuances as well as underdiagnosing diseases with Th2 phenotypes are limitations when generating PMAPs and interrogating the PMaE. A third limitation is that, as noted previously, the literature does contain some contradictory evidence regarding the authors’ hypothesis, such that Th2 dominance is suggested by some studies to be protective rather than permissive for cancer progression.

It is also important to remember that not all disease-treatment combinations conform to the pattern described herein. Section 2.1.3 provides examples in which elements of the Th2 system of cells and cytokines provided important factors that increased treatment effectiveness in specific settings.

Additional unresolved questions remain on this topic. For example, it is yet to be established that a regime of PMAP-driven interventions can influence a patient’s PMaE and TME to produce quantifiably better outcomes for patients with active cancer, or for those who may be in early or surveillance only stages where cancer is indolent. Future research with properly designed studies is needed to address these important and impactful questions.

## 8 Conclusion

The authors present here a biologically plausible, testable conceptual framework. Substantial evidence supports the component elements of the authors’ hypothesis, including reciprocal inhibition between Th1 and Th2 immunity; favorable and unfavorable effects on the TME characterized by Th1 and Th2 immunity respectively, including cross-talk between tumor and Th2/Treg cell systems; the effects of the PMaE on the TME; the Th2 polarizing effects of specific exposomic and other comorbidities; and the effects of TCA breaks and mutations on oncometabolite generation and M1 macrophage polarization. Demonstrating that the associations between the elements of this framework reflect causal connections is a matter for future studies.

The existing body of evidence and the framework assembled by the authors provides a background for the authors’ hypothesis that, because TCA breaks generate oncometabolites but also drive the macrophage M1 phenotype, the normal case when TCA breaks occur is a shift to M1 to activate Th1 immunity, promoting clearance of cancer cells generated by the oncometabolite production. However, if genomic, exposomic, or other factors drive sufficient Th2 dominance in the PMaE and TME, the shift to M1/Th1 normally promoted by the TCA breaks will instead be inhibited and thus there will not be sufficient Th1 system activation to provide anti-cancer surveillance. The result is a greater likelihood of cancer expression in the Th2 dominant patient. This is a testable hypothesis.

The authors further assert that a PMAP-based evaluation of the PMaE focused on factors known to drive Th2 dominance and other factors relevant to PMaE and TME status would provide a method for identifying and modifying key contributors to a Th2 dominant PMaE, improving cancer patient prognosis, reducing risk for those at higher cancer risk, and reducing risk of recurrence in cancer survivors. Additionally, controlling for PMaE and TME status in clinical trial design may help avoid false negative study outcomes. The development of a patient macroenvironment profile (PMAP) that focuses on quantifiable immunological, metabolic, exposomic, and other related factors would be a useful tool in advancing this work. These are likewise testable hypotheses that are suitable topics for future research.

The hypotheses presented here represent a novel assembly of known connections between established concepts in the cancer literature, with implications for a novel evaluative tool that may provide substantially increased understanding of patient-specific influences on cancer outcomes and may provide a method for incrementally improving outcomes of existing treatment methods.
